# Patrilineal Perspective on the Austronesian Diffusion in Mainland Southeast Asia

**DOI:** 10.1371/journal.pone.0036437

**Published:** 2012-05-07

**Authors:** Jun-Dong He, Min-Sheng Peng, Huy Ho Quang, Khoa Pham Dang, An Vu Trieu, Shi-Fang Wu, Jie-Qiong Jin, Robert W. Murphy, Yong-Gang Yao, Ya-Ping Zhang

**Affiliations:** 1 State Key Laboratory of Genetic Resources and Evolution, Kunming Institute of Zoology, Chinese Academy of Sciences, Kunming, China; 2 School of Life Sciences, University of Science and Technology of China, Hefei, China; 3 KIZ/CUHK Joint Laboratory of Bioresources and Molecular Research in Common Diseases, Kunming, China; 4 Laboratory for Conservation and Utilization of Bio-resource, Yunnan University, Kunming, China; 5 Department of Immunology, Hanoi Medical University, Hanoi, Vietnam; 6 Key Laboratory of Animal Models and Human Disease Mechanisms, Kunming Institute of Zoology, Chinese Academy of Sciences, Kunming, China; 7 Centre for Biodiversity and Conservation Biology, Royal Ontario Museum, Toronto, Canada; 8 Graduate University of the Chinese Academy of Sciences, Beijing, China; Erasmus University Medical Center, The Netherlands

## Abstract

The Cham people are the major Austronesian speakers of Mainland Southeast Asia (MSEA) and the reconstruction of the Cham population history can provide insights into their diffusion. In this study, we analyzed non-recombining region of the Y chromosome markers of 177 unrelated males from four populations in MSEA, including 59 Cham, 76 Kinh, 25 Lao, and 17 Thai individuals. Incorporating published data from mitochondrial DNA (mtDNA), our results indicated that, in general, the Chams are an indigenous Southeast Asian population. The origin of the Cham people involves the genetic admixture of the Austronesian immigrants from Island Southeast Asia (ISEA) with the local populations in MSEA. Discordance between the overall patterns of Y chromosome and mtDNA in the Chams is evidenced by the presence of some Y chromosome lineages that prevail in South Asians. Our results suggest that male-mediated dispersals via the spread of religions and business trade might play an important role in shaping the patrilineal gene pool of the Cham people.

## Introduction

The Austronesian language family is one of the largest and most widespread language families. It is spoken by more than 350 million people on islands from Madagascar to Easter Island [1], [2]. Nevertheless, the languages in this family have a rather limited distribution on the mainland. Chamic, the representative language of the family, is spoken by the Cham people. In Mainland Southeast Asia (MSEA), Chamic exists as a “linguistic enclave”, because it is surrounded by non-Austronesian-speaking groups (e.g. Mon-Khmers) [3], [4], [5]. Many studies investigate the diffusion of Austronesian in MSEA by tracing the origin of the Cham people. The “Out-of-Taiwan” hypothesis regards the Cham ancestors as the Austronesian immigrants from Island Southeast Asia (ISEA) and immigration is dated to around 500 BC [4], [6], [7]. Before the arrival of the Austronesian immigrants, southern Vietnam appears to have been occupied by the local Austro-Asiatic speakers, especially Mon-Khmers [8]. There is a high chance of admixture between the Chams and Mon-Khmer groups. Previously linguistic analyses of the Chamic report that some loan-words from Mon-Khmer languages form indigenous cultural contributions [4], [6]. The “Nusantao Maritime Trading and Communication Networks” hypothesis states that cultural diffusion through trading and communication networks played an important or even dominant role in the ethnogenesis of the Cham [9]. Because the origin of the Cham people is open to debate, the demographic history of the Austronesians in Southeast Asia requires further investigation.

**Figure 1:**
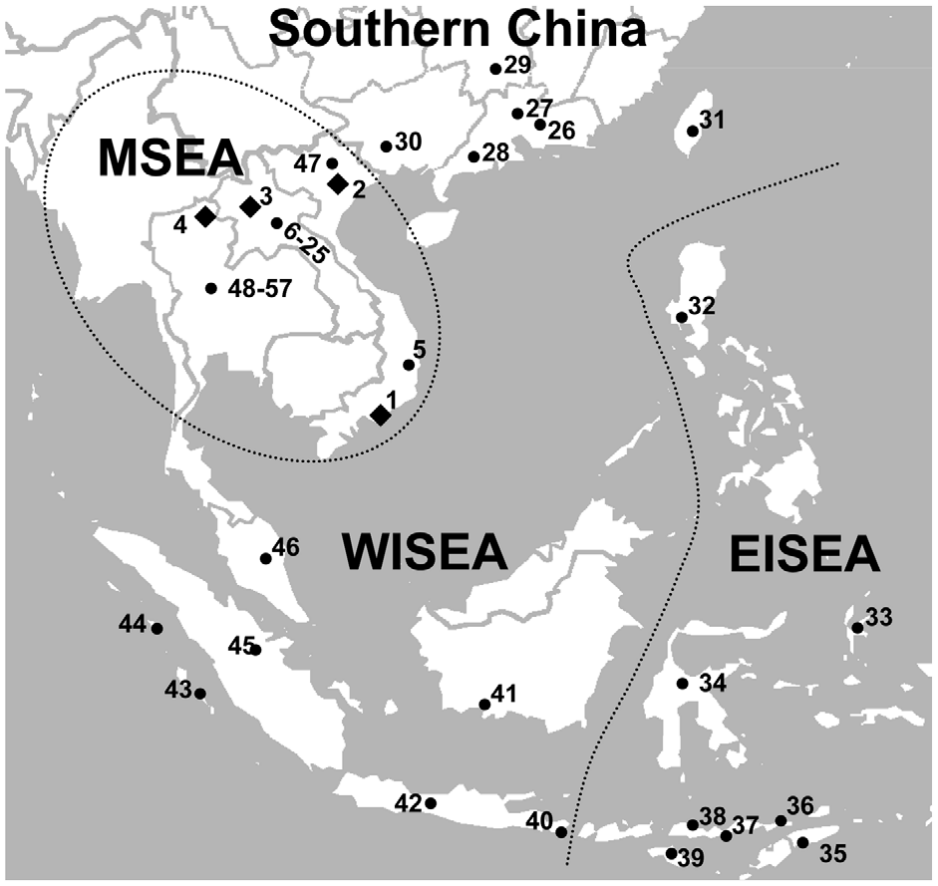
Populations from southern China and Southeast Asia analyzed in this study. Diamonds denote the location of populations newly sampled in this study; populations reported are indicated by bold circles. Population ID numbers are shown in Table 1. MSEA, Mainland Southeast Asia; WISEA, western Island Southeast Asia; EISEA, eastern Island Southeast Asia.

Analyses of mitochondrial DNA (mtDNA) variation of the Cham population resolve a closer relationship with populations in MSEA rather than with those from ISEA, and this occurs despite that recent gene flow from ISEA [10]. This result suggests that the origin of the Cham people likely involves the massive assimilation of local Mon-Khmer populations, and this is accompanied with language shift. Thus, the Austronesian diffusion in MSEA appears to mediated mainly by cultural diffusion [10]. Because mtDNA data only offer a maternal perspective, only half of the story is known. Does patrilineal history reveal the same story? We address this question by evaluating non-recombining region of the Y chromosome (NRY) markers, including 26 single-nucleotide polymorphisms (Y-SNPs) and eight short tandem repeats (Y-STRs), in 59 male Cham individuals whose matrilineal histories are known [10]. For comparison, the NRY markers of 76 Kinh, 25 Lao, and 17 Thai males were also surveyed (Figure 1; Table 1).

**Table 1:** General information for 57 populations in southern China and Southeast Asia.

No.	Group	Population	Size	Language	Location	Y-SNP	Y-STR	Reference
1	MSEA	Cham¶	59	Austronesian	Binh Thuan, Vietnam	+	+	This Study
2	MSEA	Kinh¶	76	Austro-Asiatic	Hanoi, Vietnam	+	+	This Study
3	MSEA	Lao	25	Tai-Kadai	Luang Prabang, Laos	+	+	This Study
4	MSEA	Thai	17	Tai-Kadai	Northern Thailand	+	+	This Study
5	MSEA	Vietnam	70	Austro-Asiatic	Southern Vietnam	+	+	[27] §
6	MSEA	Aheu	38	Austro-Asiatic	Laos	+	+	[33]
7	MSEA	Alak	31	Austro-Asiatic	Laos	+	+	[33]
8	MSEA	Bit	28	Austro-Asiatic	Laos	+	+	[33]
9	MSEA	Bo	28	Austro-Asiatic	Laos	+	+	[33]
10	MSEA	Brau	32	Austro-Asiatic	Laos	+	+	[33]
11	MSEA	Inh	33	Austro-Asiatic	Laos	+	+	[33]
12	MSEA	Jeh	32	Austro-Asiatic	Laos	+	+	[33]
13	MSEA	Kataang	38	Austro-Asiatic	Laos	+	+	[33]
14	MSEA	Katu	45	Austro-Asiatic	Laos	+	+	[33]
15	MSEA	Khmu	51	Austro-Asiatic	Laos	+	+	[33]
16	MSEA	Lamet	35	Austro-Asiatic	Laos	+	+	[33]
17	MSEA	Laven	49	Austro-Asiatic	Laos	+	+	[33]
18	MSEA	Mal	50	Austro-Asiatic	Laos	+	+	[33]
19	MSEA	Ngeg	35	Austro-Asiatic	Laos	+	+	[33]
20	MSEA	Oy	50	Austro-Asiatic	Laos	+	+	[33]
21	MSEA	So	49	Austro-Asiatic	Laos	+	+	[33]
22	MSEA	Suy	39	Austro-Asiatic	Laos	+	+	[33]
23	MSEA	Talieng	35	Austro-Asiatic	Laos	+	+	[33]
24	MSEA	Xinhmul	29	Austro-Asiatic	Laos	+	+	[33]
25	MSEA	Daw	51	Hmong-Mien	Laos	+	+	[33]
26	Southern China	Han	164	Sinitic	Southern China	+	+	[27] §
27	Southern China	Miao	58	Hmong-Mien	Southern China	+	+	[27] §
28	Southern China	She	51	Hmong-Mien	Southern China	+	+	[27] §
29	Southern China	Tujia	49	Tibeto-Burman	Southern China	+	+	[27] §
30	Southern China	Yao	58	Hmong-Mien	Southern China	+	+	[27] §
31	Southern China	Taiwan	48	Austronesian	Taiwan	+	+	[27] §
32	EISEA	Philippines	48	Austronesian	Philippines	+	+	[27] §
33	EISEA	Moluccas	29	Austronesian	Moluccas	+	+	[27] §
34	EISEA	Sulawesi	54	Austronesian	Sulawesi	+	+	[27] §
35	EISEA	Timor†	9	Austronesian	Timor	+	+	[27] §
36	EISEA	Alor	27	Austronesian	Alor	+	+	[27] §
37	EISEA	Lembata	89	Austronesian	Lembata	+	+	[27] §
38	EISEA	Flores	388	Austronesian	Flores	+	+	[27] §
39	EISEA	Sumba	349	Austronesian	Sumba	+	+	[27] §
40	WISEA	Bali	634	Austronesian	Bali	+	+	[27] §
41	WISEA	Borneo	85	Austronesian	Borneo	+	+	[27] §
42	WISEA	Java	61	Austronesian	Java	+	+	[27] §
43	WISEA	Mentawai	74	Austronesian	Mentawai	+	+	[27] §
44	WISEA	Nias	60	Austronesian	Nias	+	+	[27] §
45	WISEA	Sumatra	37	Austronesian	Sumatra	+	+	[27] §
46	WISEA	Malay	32	Austronesian	Malaysia	+	+	[27] §
47	MSEA	Vietnam2	48	Austro-Asiatic	Hanoi, Vietnam	+*	+	[11]
48	MSEA	Thai2	40	Tai-Kadai	Thailand	+*	+	[11]
49	MSEA	Mon	15	Austro-Asiatic	Lamphum, Thailand	-	+	[12] §
50	MSEA	Lawa	50	Austro-Asiatic	Chiang Mai, Thailand	-	+	[12] §
51	MSEA	Paluang	23	Austro-Asiatic	Chiang Mai, Thailand	-	+	[12] §
52	MSEA	Blang	40	Austro-Asiatic	Chiang Rai, Thailand	-	+	[12] §
53	MSEA	H'tin	40	Austro-Asiatic	Nan, Thailand	-	+	[12] §
54	MSEA	Yuan	92	Tai-Kadai	Chiang Mai, Thailand	-	+	[12] §
55	MSEA	Lue	96	Tai-Kadai	Northern Thailand	-	+	[12] §
56	MSEA	Khuen	29	Tai-Kadai	Chiang Mai, Thailand	-	+	[12] §
57	MSEA	Yong	31	Tai-Kadai	Lamphum, Thailand	-	+	[12] §

Note:

¶ genomic DNA was extracted and purified at the laboratory of the Immunophysiopathology Department, Hanoi Medical University;

§ requests for the data access could be directed to the authors;

† Timor was excluded in PCA and MDS analyses because of fewer sample size;

* populations were genotyped with the lower Y-SNPs resolution, and were not considered in PCA.

## Results

### Phylogeny of Y chromosomes

**Figure 2:**
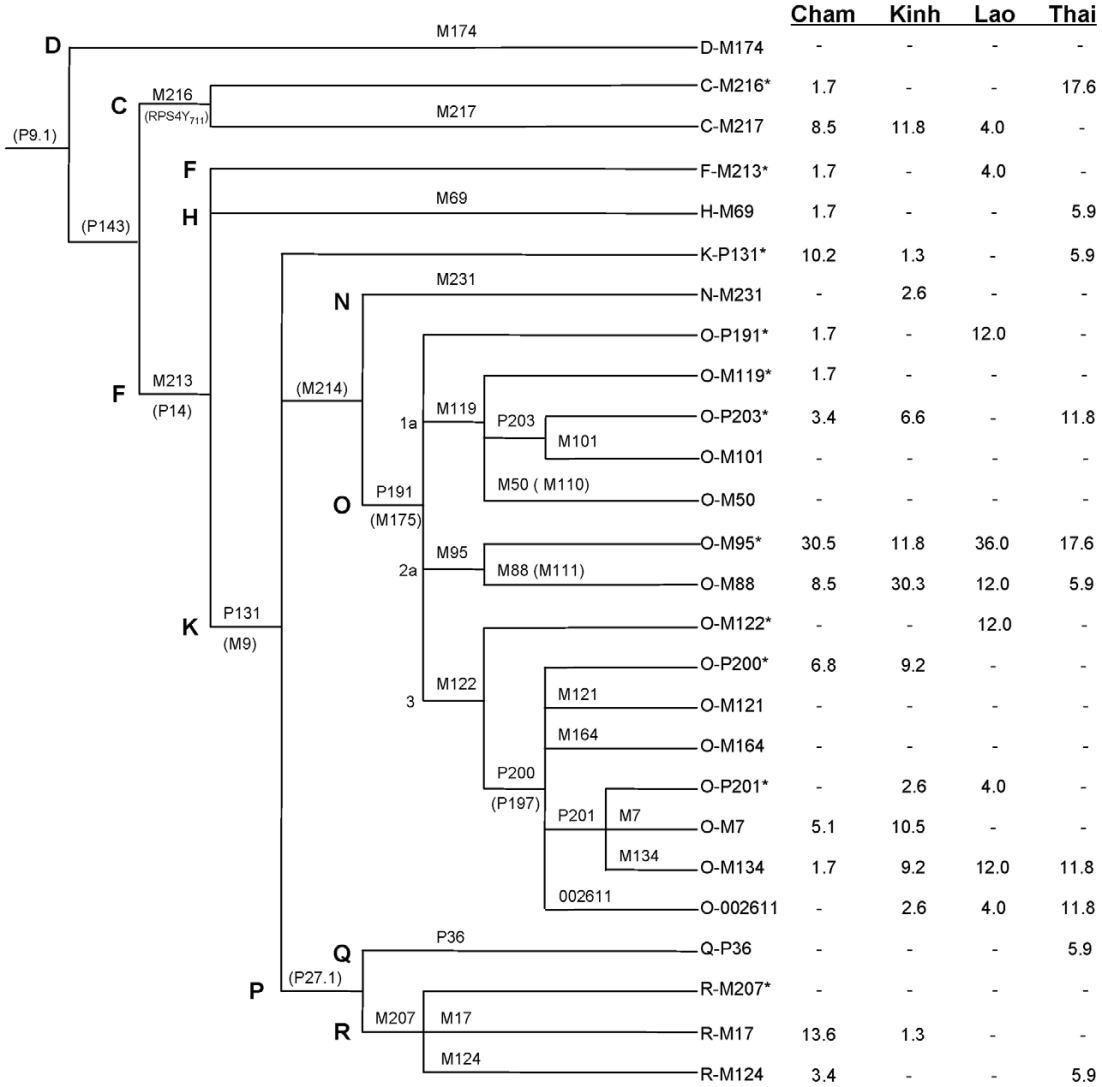
Classification tree of 26 NRY haplogroups along with their frequencies (%) in four populations. Haplogroup defining markers are given along the branches; corresponding markers genotyped in Karafet et al. [27] are noted in brackets. The names of haplogroups are shown to the right of the branches using the mutation-based nomenclature of the Karafet et al. [35].

Based on 26 Y-SNPs, all 177 newly genotyped males from the four populations were assigned to specific (sub-)haplogroups (paragroups) defined in the phylogeny (Figure 2; Table S1). Nearly 60% of the Chams' Y chromosomes belonged to P191-derived haplogroups. Within this group, O-M95* predominated and accounted for around 30% of all samples. Haplogroup C-M216, consisting of C-M217 and C-M216*, comprised 10.2% of the patrilineal lineages. One Cham individual (∼1.7%) rooted near the base of the tree as haplogroup F-M213* and six individuals (∼10.2%) rooted at the base of the tree as haplogroup K-P131*. Notably, South Asian-prevailing haplogroups R-M17 (∼13.6%), R-M124 (∼3.4%), and H-M69 (∼1.7%) are identified with the Chams.

**Figure 3:**
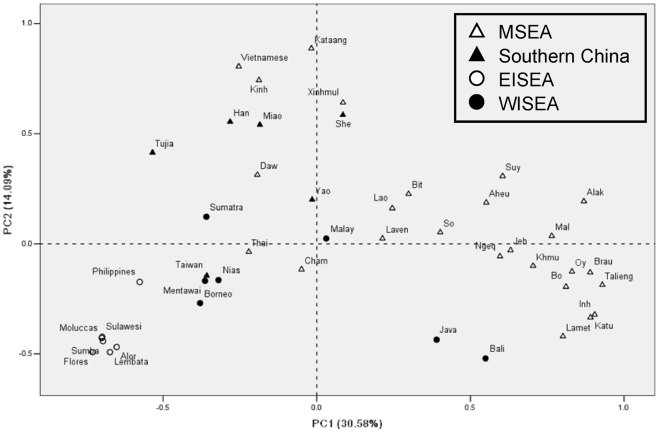
PCA plot based on NRY haplogroup frequencies of 45 populations in southern China and Southeast Asia.

**Figure 4:**
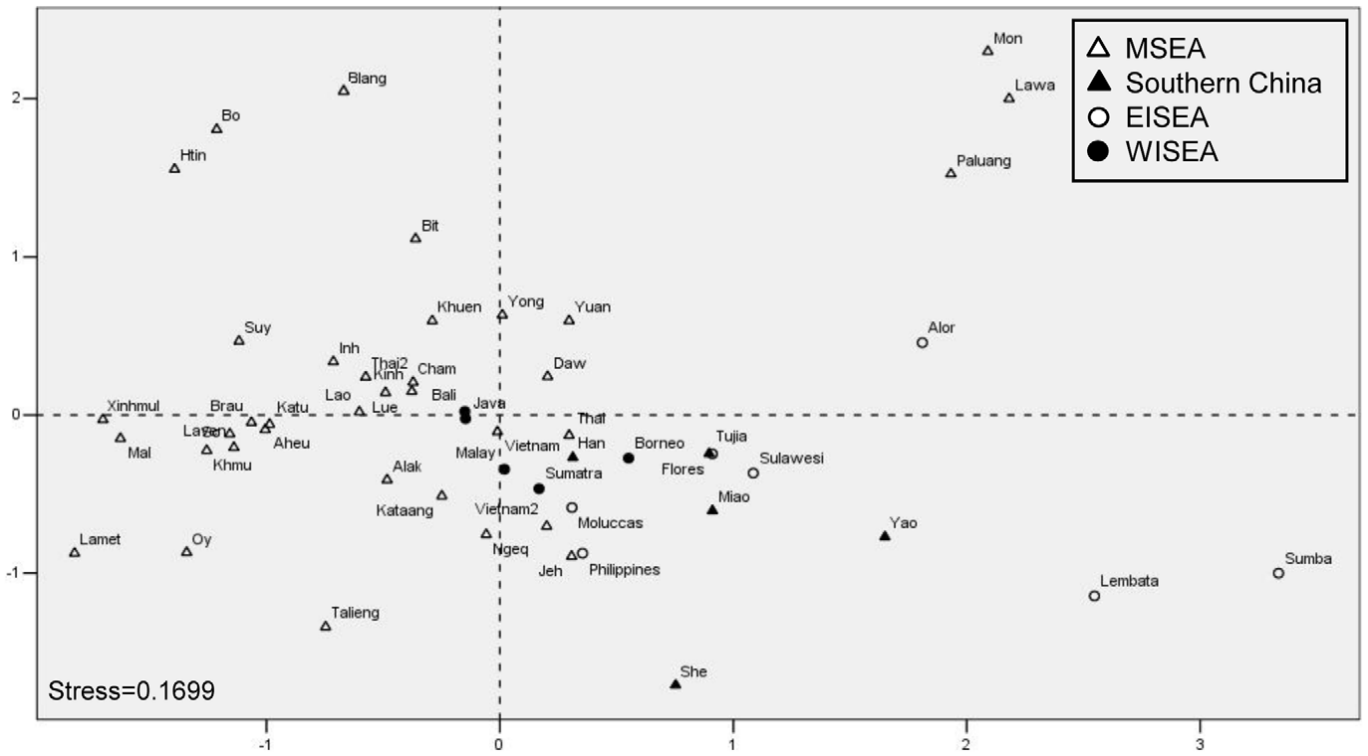
MDS plot of 53 populations with *R*_ST_ genetic distances based on eight common Y-STRs. For population information, see Table 1. Because of severe genetic drift [27], populations Taiwan, Nias, and Mentawai that were resolved as the outliers in the initial analyses and were excluded.

### Population structure

Genetic relationships between the Cham and other Southeast Asian populations were discerned with the aid of additional published Y-chromosomal datasets (Figure 1; Table 1). We employed a principal component analysis (PCA) based on the NRY haplogroup distribution frequencies of 45 populations (Table S2) to show the overall clustering pattern of the populations. Populations from eastern ISEA (EISEA) and from Laos formed two clusters in the first PC (Figure 3) and this pattern was mainly owed to haplogroups C-M216, K-P131*, and O-M95* (Figure S1). The second PC resolved a close affinity between the Kinh and Vietnamese (most likely, the Kinh) populations with those from mainland southern China due to the high frequency of haplogroup O-M88 (Figure S1). The Cham population showed a close affinity to some but not all populations from western ISEA (WISEA; Figure 3). The clustering pattern revealed by PC1 and PC2 was statistically significant (*P*<0.05) in AMOVA based on the same profiles of haplogroup distribution frequencies (Table S2). Nevertheless, in terms of the linguistic affinities, the difference between Austronesian (i.e. Cham and WISEA populations) and non-Austronesian (i.e. other MSEA populations) was not statistically significant according to AMOVA (*p* = 0.08). We incorporated data for eight common Y-STRs (DYS19, DYS389-I, DYS389-II, DYS390, DYS391, DYS392, DYS393, and DYS439) from additional populations in MSEA [11], [12], Multidimensional scaling (MDS) based on *R*_ST_ genetic distances for these Y-STRs did not associate the Chams with populations from WISEA (Figure 4).

**Figure 5:**
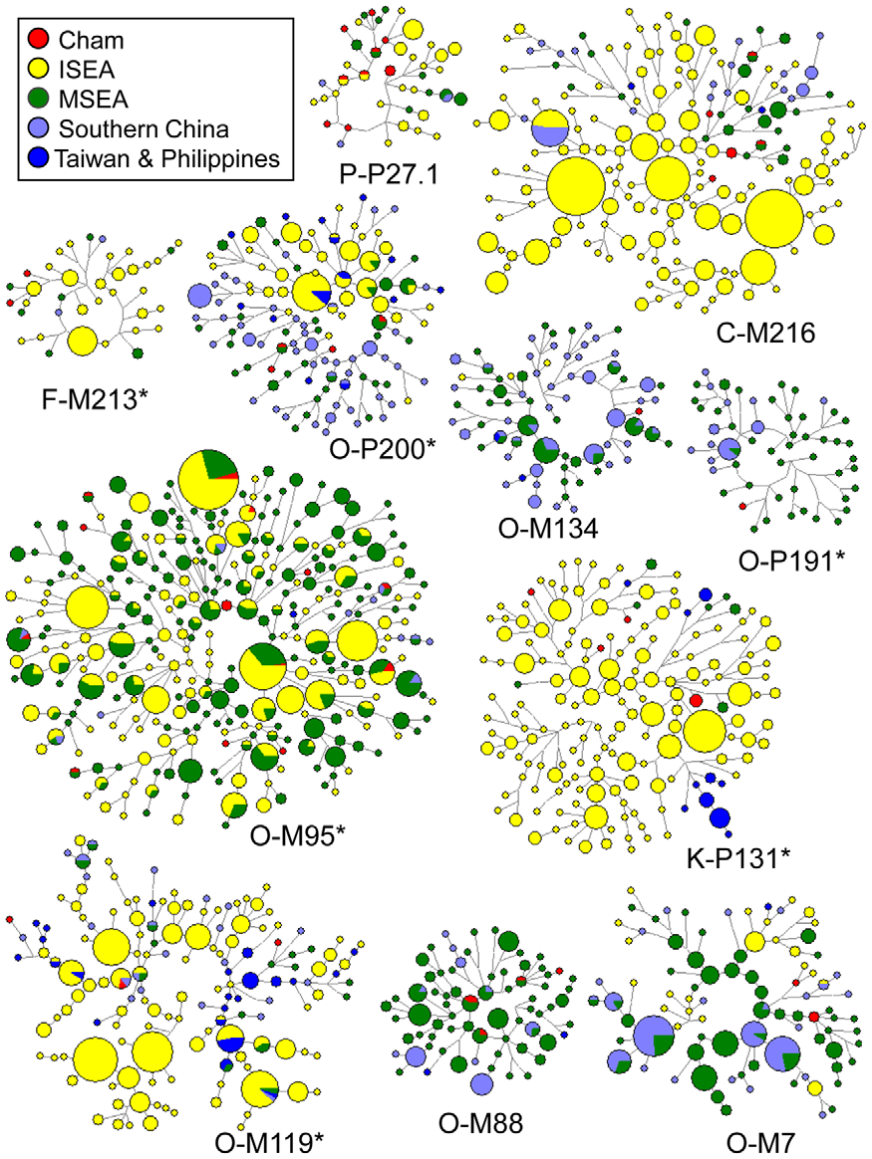
Median-joining networks of eight Y-STRs within NRY haplogroups C-M216, F-M213*, K-P131*, O-M7, O-M88, O-M95*, O-M119*, O-M134, O-P191*, O-P200*, and P-P27.1. Sizes of the circles are proportional to haplotypes frequencies. The lengths of the lines are proportional to the mutational steps.

**Figure 6:**
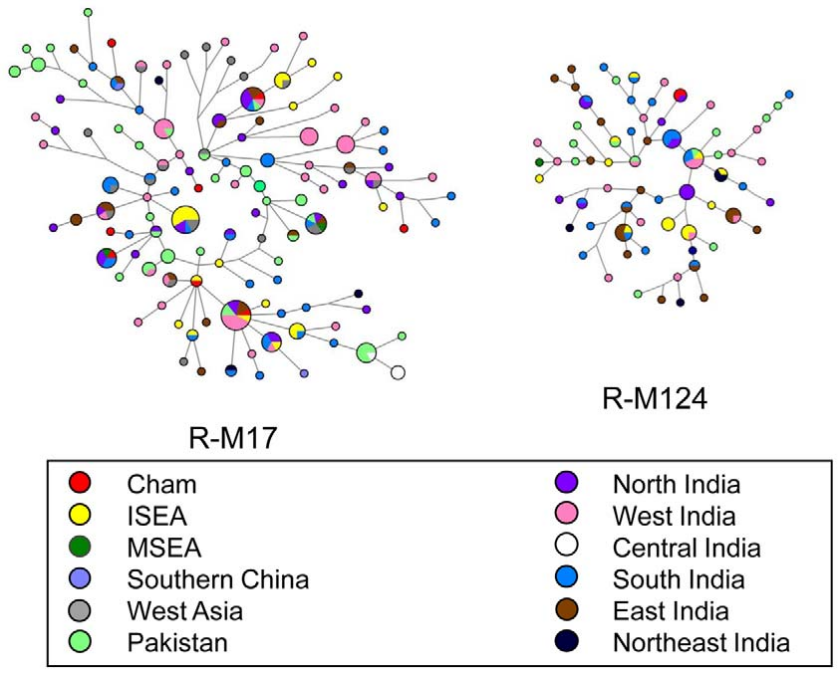
Median-joining networks of eight Y-STRs within NRY haplogroups R-M17 and R-M124. Sizes of the circles are proportional to haplotypes frequencies. The lengths of the lines are proportional to the number of mutational steps.

**Figure 7:**
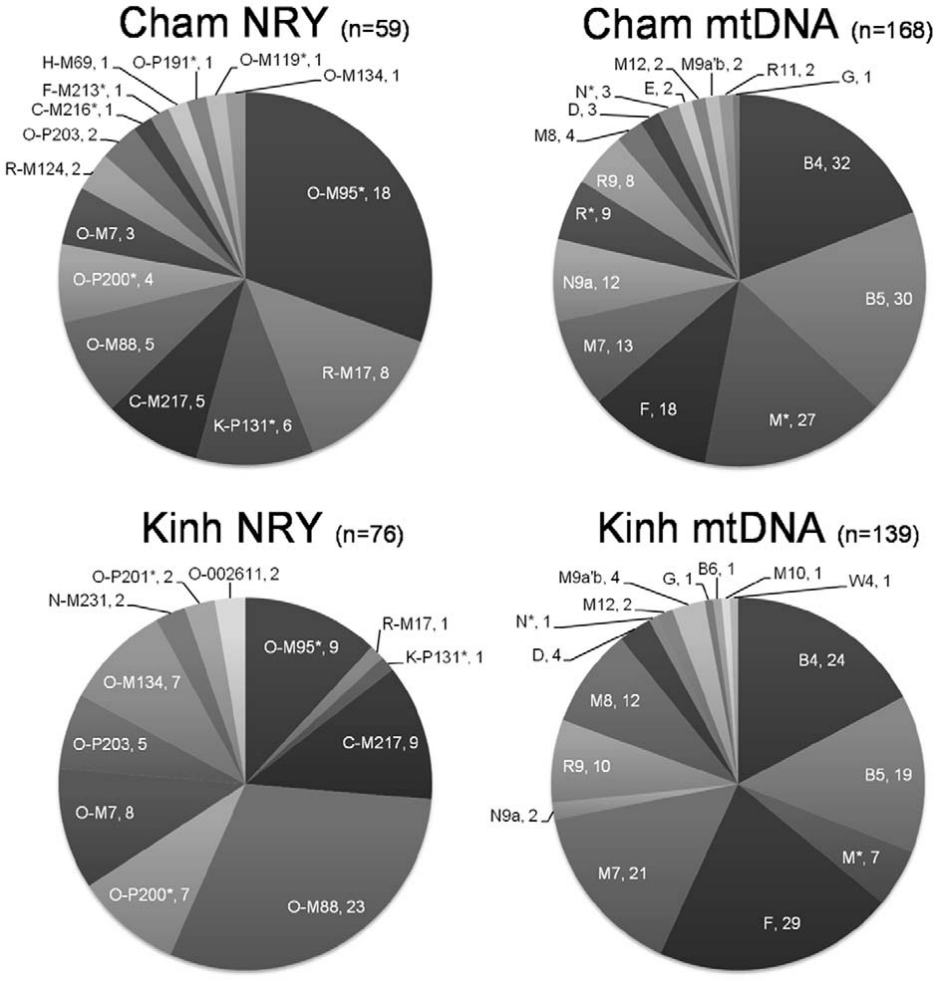
NRY and mtDNA haplogroup profiles for the Chams and the Kinhs. For mtDNA haplogroups, M* includes M17, M20, M21d, M22, M33c, M50, M51, M71, M72, M73, and M77; N* includes N21 and N23; R* includes R22 and R23 [10].

### Admixture in the Cham population

The origin of the Chams could not be simply explained as a demic diffusion of Austronesian immigrants from WISEA. The genetic patterns between the Cham and other Southeast Asian populations, as detected in PCA and MDS, suggested a more complex history. The complex demographic process likely involved genetic admixture with local non-Austronesian speakers in MSEA. Therefore, we performed the admixture analysis [13], [14] to quantify the proportion of genetic contribution from WISEA and MSEA to the Chams (Table 2). The patrilineal contribution from WISEA to the Chams (0.37595) was less than that from MSEA (0.62405). Comparatively, the Vietnamese (most likely, the Kinh) population from southern Vietnam had a dominant proportion of the MSEA contribution (0.842972; Table 2), although the large standard deviation values made the results should be treated with caution.

**Table 2:** Admixture analysis of the two populations from southern Vietnam.

Admixed populations	Parental populations	
	MSEA (n = 890)	WISEA (n = 983)
Cham (n = 59)	0.62405¶0.629437±0.256634†	0.375950.370563±0.256634
Vietnamese (n = 70)	0.8429720.839953±0.56035	0.1570280.160047±0.56035

Note:

¶ admixture coefficient;

† bootstrap average and standard deviation of the admixture coefficient were obtained by bootstrap with 1000 replications.

### Haplotype diversity analyses

To discern the relationship between the Y-STR haplotypes in the Chams and other Southeast Asians, median-joining networks [15] were constructed using eight common Y-STRs for each of the 11 haplogroups found in the Cham population (Figure 5). In the networks of haplogroups O-M95* and P-P27.1, some haplotypes were exclusively shared between the Cham and ISEA populations. In the networks of C-M216, F-M213*, and K-P131*, some haplotypes in the Chams were derived directly from those in ISEA populations. These lineages in the Chams were most likely introduced by recent gene flow from ISEA. In contrast, the networks for haplogroups O-M7, O-M88, O-M134, O-P191*, and O-P200* indicated closer associations between the Chams and MSEA populations. Most Cham lineages either had identical counterparts or were linked to those haplotypes in MSEA populations; the numbers of mutations between the Chams and MSEA were less than those between the Chams and ISEA (Table S3). These patterns would suggest that these Chams lineages had an *in situ* origin from MSEA. Among the 48 haplotypes identified in the Chams, 11 and 18 were shared with those in ISEA and MSEA, respectively (Table S3). Nevertheless, the counts for shared haplotype did not differ significantly (two-tailed Fisher's exact test, *P* = 0.303; Table S4). Moreover, six haplotypes belonging to haplogroup O-M95* were shared by both ISEA and MSEA groups. The exact origin of these lineages in the Chams remains elusive.

To trace the source of the exotic South Asian prevailing components, we incorporated published data (Table S5) from India [16], [17], Pakistan [16], and West Asia [18], [19] and reconstructed median-joining networks of haplogroups R-M17 and R-M124 (Figure 6). All haplotypes in the Chams were scattered in the networks, which implied that these lineages had an origin via recent gene flow rather than deeply rooted ancestry. Two Cham lineages of R-M124 were shared the same haplotype with those from North India. This observation suggested that North India might be the original source of the R-M124 lineages in the Chams. The relationships among lineages of R-M17 were complex in the network, which suggested multiple geographic/ethnic sources for the R-M17 lineages in the Chams.

## Discussion

Integrating the information from two uniparentally inherited markers (NRY and mtDNA) is a powerful means of disentangling the human population histories [20], and especially for elucidating sex-biased migrations and social-cultural effects [21]. Compared with our previous study for mtDNA variation in the Chams [10], the current assessment for NRY variation facilitates a better understanding into the origin of the Cham people. Both NRY and mtDNA haplogroup profiles (Figure 7) suggest that, in general, the Chams are indigenous to Southeast Asia. Characteristic East and Southeast Asian lineages, viz., NRY haplogroups O-P191 and C-M217, together with mtDNA haplogroups B, F, M7, and R9, accounted for the majority of the patrilineal (∼67.8%) and matrilineal (∼60.1%) gene pools of the Chams, respectively. Some ancient Southeast Asian components (NRY haplogroups: C-M216*, F-M213*, and K-P131*; mtDNA haplogroups: M*, N*, and R*) were also identified in the Chams.

The origin of the Chams appears to be much more complex, at least based on the results of PCA, MDS, AMOVA, and haplotype (near-) matching analyses. Recent gene flow from ISEA is detected in the patrilineal pool of the Chams, most likely via the dispersal of Austronesian speakers. Further, the Cham population also contains a significant amount of local genetic contributions from non-Austronesian populations in MSEA. This pattern corresponds with our previous study based on mtDNA [10]. Taken together, the origin of the Chams is mainly a result of admixture between the Austronesian immigrants from ISEA with the indigenous populations (most likely, Mon-Khmers) in MSEA.

South Asian NRY haplogroups R-M17, R-M124, and H-M69 [16], [22] are common in the Chams (∼18.6%; Figures 2) yet no mtDNA haplotypes are known [10]. Male South Asians contribute to the genetic makeup of Chams, but not South Asian females. The existence of these South Asian patrilineal lineages was in good accordance with the archaeological and historical records. The dominant religion of the Cham people is known to have been Hinduism (overwhelmingly Shaivism) and their culture was deeply influenced by that of India [3], [7]. Both Indian and Cham people appear to have played important roles in Southeast Asian maritime trade [23], [24]. Contact between the two peoples makes gene flow between them inevitable. The discordance between NRY and mtDNA contributions in the Chams (Figure 7) is well explained by the male-mediated dispersals, most likely through the spread of religions and business trade. In particular, the admixture between alien males and local females is compatible with the matrilocal residence in the Cham people [25], [26].

Patrilineal genetic structuring differs between the Chams and Kinhs. For instance, in contrast to the Chams, frequently the Kinhs have lineages (8/76, ∼10.5%) from the characteristic Chinese haplogroup O-M7 [27] yet only one lineage from the South Asian haplogroup R-M17 (Figure 7). In addition to the Sinicized cultures, substantial Chinese assimilation into the Kinh people via immigration is suggested for northern Vietnam [3], [7]. Thus, the different ethnohistories of the Chams and Kinhs are reflected by their unique mtDNA and NRY patterns.

In summary, this study expands our knowledge on the complex history of the Austronesian diffusion in MSEA. Further improvements to the resolution of the NRY tree [20], [28] will help to unravel the story of the Cham people. This initiative will also benefit from the employment of genome-wide autosomal markers [29], [30], [31]. In the future, a comprehensive study involving extensive sampling will pinpoint more details about the demographic history, such as the source and route for migration, the timing for admixture and expansion.

## Materials and Methods

### Samples and data collection

Blood samples of 177 unrelated males were collected from four populations (Table 1; Figure 1). Among them, samples from 59 Cham individuals were collected from Binh Thuan province, southern Vietnam. Binh Thuan was part of the Cham principality of Panduranga, the last Cham territory that had been annexed by Nguyen Vietnam in 1832 AD [3], [7], and it was said to harbor a significant number of Chamic speakers [7]. The mtDNA data of the Cham, Kinh, and Thai populations were previously reported [10], [32]. This study was approved by the Institutional Review Board of Kunming Institute of Zoology. All subjects were interviewed to obtain informed written consent before sample collection.

Comparative NRY data from southern China and Southeast Asia (Figure 1; Table 1) were taken from previously published literature [11], [12], [27], [33]. We uniformed all Y-SNPs and Y-STRs data into the same resolution to include as more populations as possible. This truncation of some data caused the NRY haplogroups collapsed into 16 clusters (Table S2), and Y-STRs were reduced to eight loci (DYS19, DYS389-I, DYS389-II, DYS390, DYS391, DYS392, DYS393, and DYS439). Additional data of haplogroups R-M17 and R-124 were collected from published South and West Asian datasets [16], [17], [18], [19] (Table S5).

### DNA extraction and genotyping

Genomic DNA was extracted by the standard phenol/chloroform methods. Seventeen Y-SNPs (Table S1) were genotyped by the GenomeLab^TM^ SNPstream® (Beckman Coulter). We used three panels of multiplex PCR reactions following manufacturer's recommendation (Protocol S1). The primers for multiplex PCR and single base extension reactions were designed by Autoprimer software (Beckman Coulter) [34]. To improve the resolution of phylogeny, we further screened nine Y-SNPs by direct sequencing some individuals (Table S1). The PCR amplification and sequencing primers were previously reported [35]. Using described methods [36], [37], [38], we genotyped eight Y-STRs (DYS19, DYS389-I, DYS389-II, DYS390, DYS391, DYS392, DYS393, and DYS439) on an ABI 3730 DNA Analyzer (Applied Biosystems). For DYS389-I and DYS389-II, we used the genotyped data of DYS389-I, and DYS389-II minus DYS389-I in our analyses.

### Data analysis

Arlequin 3.5 (http://cmpg.unibe.ch/software/arlequin35/) was used to calculate AMOVA and *R*_ST_ distances [39]. Principal component analysis (PCA) and multidimensional scaling (MDS) were performed using SPSS 13.0 software (SPSS). In PCA, the original haplogroup frequency data were transformed to standardize against the different effect of genetic drift on haplogroups of different frequencies [40]. Admix 2.0 (http://web.unife.it/progetti/genetica/Isabelle/admix2_0.html) was used to estimate the level of admixture of MSEA and WISEA groups in the Cham and Vietnamese populations [13], [14]. The average haplogroup frequencies of MSEA and WISEA were taken for the two parental populations, respectively. Median-joining networks [15] of Y-STRs within certain haplogroups were constructed with NETWORK 4.6 (http://www.fluxus-engineering.com/network_terms.htm).

## Supporting Information

Figure S1 **Plot of haplogroup contribution of the first and second PC.** The contribution of each haplogroup is calculated as the factor scores for PC1 and PC2 with regression (REGR) method in SPSS 13.0 software (SPSS).
(TIF)

Table S1 **The variations of Y-SNPs and Y-STRs in 177 unrelated males analyzed in this study.**
(XLS)

Table S2

Table S3

Table S4 **Fisher's exact test for haplotype (near-) matching analysis.**
(DOC)

Table S5 **General information of additional populations analyzed in haplogroups R-M17 and R-M124.**
(XLS)

Protocol S1 **The primers and protocols for the SNPs genotyped by the GenomeLab^TM^ SNPstream®.**
(DOC)
