# Supplementary figures and images for: Patrilineal Perspective on the Austronesian Diffusion in Mainland Southeast Asia
Source: PLoS One. 2012 May 7;7(5):e36437. doi: 10.1371/journal.pone.0036437 (PMC3346718; doi:10.1371/journal.pone.0036437)

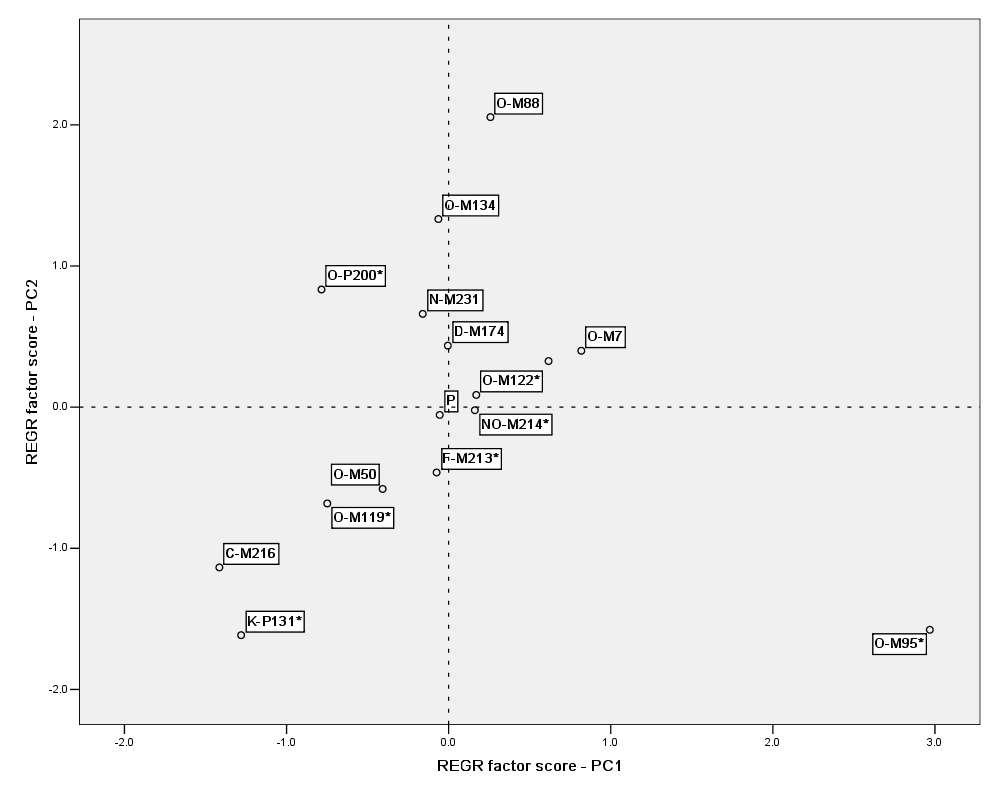

Supplement: Figure S1 — Plot of haplogroup contribution of the first and second PC. The contribution of each haplogroup is calculated as the factor scores for PC1 and PC2 with regression (REGR) method in SPSS 13.0 software (SPSS). (TIF) [file pone.0036437.s001.tif]
