# Supplementary material for: Patrilineal Perspective on the Austronesian Diffusion in Mainland Southeast Asia
Source: PLoS One. 2012 May 7;7(5):e36437. doi: 10.1371/journal.pone.0036437 (PMC3346718; doi:10.1371/journal.pone.0036437)
Supplement: Table S4 — Fisher's exact test for haplotype (near-) matching analysis. (DOC) [file pone.0036437.s005.doc]

**Table S4 Fisher’s exact test for haplotype (near-) matching analysis.**

| Distance to the haplotype* | Sample number | | 2-tail *P*-value  (Fisher’s exact test) |
| --- | --- | --- | --- |
|  | MSEA | ISEA |  |
| 0 | 18 | 11 | 0.303 |
| 1 | 20 | 14 | 0.439 |
| 2 | 8 | 7 | 1.000 |
| 3 | 2 | 9 | 0.057 |
| >3 | 11 | 17 | 0.303 |
| 0+1 | 38 | 25 | 0.214 |
| 0+1+2 | 46 | 32 | 0.305 |
| 3+(>3) | 13 | 26 | 0.067 |

Note: * the distance was recorded as number of mutation steps in Y-STRs (Table S3).
