# Supplementary material for: Patrilineal Perspective on the Austronesian Diffusion in Mainland Southeast Asia
Source: PLoS One. 2012 May 7;7(5):e36437. doi: 10.1371/journal.pone.0036437 (PMC3346718; doi:10.1371/journal.pone.0036437)
Supplement: Protocol S1 — The primers and protocols for the SNPs genotyped by the GenomeLabTM SNPstream®. (DOC) [file pone.0036437.s007.doc]

**Protocol S1**

**Protocol of the GenomeLab^TM^ SNPstream® 12-plex Genotyping System** (the volume of reagents were prepared for 450 reactions)

**Multiplex PCR Reaction:**

Primer Pool (10um each) 11.25 µl

dNTPs (10um each) 16.875 µl

10X PCR Buffer II 225 µl

MgCl_2_ (25mM stock) 450 µl

AmpliTaq Gold (5U/ul) 45 µl

ddH_2_O 601.875 µl

Total 1350 µl

2 µl of the DNA samples (10 ng/µl) was added to each well of the plate, followed by the addition of 3 µl of multiplex PCR mix. Then plate was centrifuged for 1 min at 1000 RPM

PCR conditions:

94°C for 1 min

94°C for 30 sec

55°C for 30 sec 40 cycles

72°C for 1 min

4°C for storing

**Post PCR Purification:**

Exo I (10U/ul) 90 µl

SAP (1U/ul) 448 µl

10X SAP Buffer 135 µl

ddH_2_O 667 µl

Total 1350 µl

3 µl of the purification mix was added to each well of the PCR plate and centrifuged it briefly.

Purification conditions:

37°C for 30 min

96°C for 10 min

4°C for storing

**Single Base Extension Reaction:**

Extension Dilution Buffer 1692.5 µl

Extension Primer Mix (10um each) 13.5 µl

20X Extension Mix 90 µl

DNA Polymerase 9.4 µl

ddH_2_O 1336.5 µl

Total 3150 µl

7 µl of the extension mix was added to each well of the PCR plate and centrifuged it briefly.

PCR conditions:

96°C for 3 min

94°C for 20 sec

40°C for 11 sec 46 cycles

4°C for forever

**Pre Hybridization Washing of the Hybridization Plate:**

20X Wash Buffer I 2500 µl

ddH_2_O 47500 µl

Total 50000 µl

20 µl of the diluted wash buffer I was added to each well of the hybridization plate. The plate was reversed on a clean paper, flung on the sink and centrifuged at 150 RCF for 1 min. This step was repeated twice.

**Preparation of Hybridization Solution:**

Hybridization Solution 3402 µl

Hybridization Additive 198 µl

Total 3600 µl

8 µl of the hybridization solution was added to each well of the PCR plate and mixed it. Then 15 µl of the mixed solution from PCR plate was added to the hybridization plate and left the plate in a moist chamber at 42°C for two hours.

**Post-Hybridization Washing of the Hybridization Plate:**

20X Wash Buffer II 781 µl

ddH_2_O 49219 µl

Total 50000 µl

20 µl of the diluted wash buffer II was added to each well of the hybridization plate. The plate was reversed on a clean paper, flung on the sink and centrifuged at 150 RCF for 1 min. This step was repeated twice. Then the glass of the hybridization plate was cleaned by using methanol.

The hybridization plate was placed in the genotyping system and the manufacturer’s recommendation was followed.

Table The information of primers in the GenomeLab^TM^ SNPstream® 12-plex Genotyping System.

| Panel | SNP | Mutation | Multiplex PCR Primer | | Single Base Extension Primer (5’-3’) |
| --- | --- | --- | --- | --- | --- |
|  |  |  | Forward Primer (5’-3’) | Reverse Primer (5’-3’) |  |
| 1 | M50 | T->C | AATACATGGGTCACTCAGTTATGTT | ATTCTTTTTTTCCGATAGGTCC | AGATAGAGTCGATGCCAGCTGCCAACAGCCTACCCAAACCACACC |
|  | M216 | C->T | TATTCATAATCACTTTTATATCCTCAACC | TAAACATGGTTCTAAATCTGAATTCTG | AGCGATCTGCGAGACCGTATAAATTCCTTTATTAAAGAAATGTAA |
|  | P131 | C->T | CCTTCCACTTAGTAACACTCAGA | ATGTAGAGAATTACATGTTGATCATTTT | CGTGCCGCTCGTGATAGAATCAATCATTCTTTGTTATCTGTGTGA |
|  | M122 | T->C | TATATTTACAGCAAACTTGGTAAACTCTAC | TTTGAGAGTCACTTGCTCTGTG | GTGATTCTGTACGTGTCGCCGAAAAGCAATTGAGATACTAATTCA |
|  | M207 | A->G | TTCAACCTCTTGTTGGAAGATT | TTAAGTTTATCAGAAGAAAGGAAAAATC | GGATGGCGTTCCGTCCTATTTTCAAAAGGTATTGTTATTCTCTTT |
|  | M101 | C->T | AGCCTGCCTGTTTGGTTC | AGGATCCCAGTTCCTGCA | ACGCACGTCCACGGTGATTTATTTAGCCAAAATATTTTTTTTTTA |
| 2 | M95 | C->T | TTGGGATCAAATGGAGTTCC | TTACATCCCTAGTAAGTCTGGACTCT | AGCGATCTGCGAGACCGTATATAAGGAAAGACTACCATATTAGTG |
|  | P201 | T->C | TGTGCTGTGCAAGTTGTGT | AGTTAAGCAATGAAGGTAGAAGGG | GGATGGCGTTCCGTCCTATTTCTTGGTTAAGTCATTTGATCTCAG |
|  | P36 | G->A | TATCCATCCATTGTGTATCTTTATCTAT | GGAGAGAGAAAAAGAGAGAGAGAGA | ACGCACGTCCACGGTGATTTCATCTATCTATCCATTATTCTCTCT |
|  | JST002611 | C->T | TGGAGTCGGAAGCCGAGG | AGTGCCTTCTGGCGCAGA | CGTGCCGCTCGTGATAGAATTCCAGAGCCCTGCTAGTAGGCACCA |
| 3 | M231 | G->A | AAAATGTGGGCTCGTTTTAAT | ACAGAAATTACAGGTATGAATTCTTTG | GCGGTAGGTTCCCGACATATAACATTTACTGTTTCTACTGCTTTC |
|  | M88 | A->G | TAAACCCAATATAGGCTATGGC | ACTATATGCTACACAGTGCTAGAGAGG | AGCGATCTGCGAGACCGTATCTTTTCTTATTCCTGCTTCTTCTGC |
|  | M134 | -1bp | ATCAAACCCAGAAGGGTTAAA | AATTCTCATTTACCACTGTGGAG | CGACTGTAGGTGCGTAACTCAAAAAGAAAAGGCCCAGGAAAGTAT |
|  | M164 | T->C | CTTGCAGAGTTGCTGTCCA | CAGGATTTTACCAAATGTTTAATATGT | ACGCACGTCCACGGTGATTTATCTATGCAATAATTAAACTGTGTA |
|  | P191 | A->G | GCAAATAGTAGCAGAAACCTTATT | TAACATTTGACATGTGGTAGGAG | CGTGCCGCTCGTGATAGAATAGAAAGGTATAGTGTTCAAAATGTA |
|  | M174 | T->C | AGACCCATCTTGCAAGGAA | AAAATGTACGTTTTTGGTTTACTCA | GGCTATGATTCGCAATGCTTCAAATGCACCCCTCACTTCTGCACT |
|  | P203 | G->A | TTTTTGAAGGCTACATGGAAAT | GTATTTTTAATTCTCACTTAGCACATATACA | GGATGGCGTTCCGTCCTATTGTTGGCTATTGAGTTAGCATAATCA |
